# Deciphering the mechanism of PSORI-CM02 in suppressing keratinocyte proliferation through the mTOR/HK2/glycolysis axis

**DOI:** 10.3389/fphar.2023.1152347

**Published:** 2023-04-07

**Authors:** Maojie Wang, Bin Tang, Huanjie Huang, Xiaodong Wu, Hao Deng, Haiming Chen, Liyan Mei, Xiumin Chen, Boudewijn Burgering, Chuanjian Lu

**Affiliations:** ^1^ The Second Affiliated Hospital, Guangzhou University of Chinese Medicine (Guangdong Provincial Hospital of Chinese Medicine), Guangzhou, China; ^2^ Molecular Cancer Research, Center of Molecular Medicine, University Medical Center Utrecht and the Oncode Institute, Utrecht, Netherlands; ^3^ Guangdong-Hong Kong-Macau Joint Lab on Chinese Medicine and Immune Disease Research, Guangzhou University of Chinese Medicine, Guangzhou, China; ^4^ State Key Laboratory of Dampness Syndrome of Chinese Medicine, The Second Affiliated Hospital of Guangzhou University of Chinese Medicine (Guangdong Provincial Hospital of Chinese Medicine), Guangzhou, China

**Keywords:** psoriasis, PSORI-CM02, proliferating keratinocytes, mammalian target of rapamycin, hexokinase 2

## Abstract

Hyperplasia of epidermal keratinocytes that depend on glycolysis is a new hallmark of psoriasis pathogenesis. Our previous studies demonstrated that PSORI-CM02 could halt the pathological progression of psoriasis by targeting inflammatory response and angiogenesis, but its effect(s) and mechanism(s) on proliferating keratinocytes remained unclear. In this study, we aim to identify components of PSORI-CM02 that are absorbed into the blood and to determine the effect(s) of PSORI-CM02 on keratinocyte proliferation and its molecular mechanism(s). We used the immortalized human epidermal keratinocyte cell line, HaCaT, as an *in vitro* model of proliferating keratinocytes and the imiquimod-induced psoriasis mouse (IMQ) as an *in vivo* model. Metabolite profiles of vehicle pharmaceutic serum (VPS), PSORI-CM02 pharmaceutic serum (PPS), and water extraction (PWE) were compared, and 23 components of PSORI-CM02 were identified that were absorbed into the blood of mice. Both PPS and PWE inhibited the proliferation of HaCaT cells and consequently reduced the expression of the proliferation marker ki67. Additionally, PPS and PWE reduced phosphorylation levels of mTOR pathway kinases. Seahorse experiments demonstrated that PPS significantly inhibited glycolysis, glycolytic capacity, and mitochondrial respiration, thus reducing ATP production in HaCaT cells. Upon treatments of PPS or PWE, hexokinase 2 (HK2) expression was significantly decreased, as observed from the set of glycolytic genes we screened. Finally, in the IMQ model, we observed that treatment with PSORI-CM02 or BPTES, an inhibitor of mTOR signaling, reduced hyperproliferation of epidermal keratinocytes, inhibited the expression of p-S6 and reduced the number of proliferating cell nuclear antigen (PCNA)-positive cells in lesioned skin. Taken together, we demonstrate that PSORI-CM02 has an anti-proliferative effect on psoriatic keratinocytes, at least in part, by inhibiting the mTOR/HK2/glycolysis axis.

## Introduction

Psoriasis (Ps) is a progressive immune-mediated skin disorder characterized by scaly, erythematous plaques on the extensor surfaces; it affects 0.1%–0.4% of the world population (Armstrong and Read, 2020; Parisi et al., 2020). To date, the dysfunction of keratinocytes, i.e., parakeratosis and hyperplasia, have been deemed the primary hallmarks in Ps pathogenesis, along with inflammatory infiltration and angiogenesis (Boehncke and Brembilla, 2018). In the context of the disease, epidermal keratinocytes proliferate in response to inflammatory stimuli and release chemokines to recruit more immune cells to the affected skin, while enriched pro-inflammatory cells are activated and react on keratinocytes by releasing cytokines such as IL-17A; this results in a self-propelling process that eventually leads to continued expansion and progression of multiple pathologic processes that characterize psoriasis (Ni and Lai, 2020; Zhou et al., 2022). Consequently, breaking this process is the primary strategy of clinical intervention. Currently, this is achieved by treatments that block the immune cell-mediated inflammatory response, such as anti-TNF-α, anti-IL-17A, and anti-IL-23 therapy, or by suppressing keratinocyte and immune cell proliferation by using methotrexate or etretinate. Despite these treatment options and our understanding of the complex molecular pathogenesis of psoriasis, the disease is still not fully curable, and therefore new drugs need to be developed.

Proliferating cells often reprogram their metabolic pathways to meet the extensive demands of ATP and biosynthetic intermediates. The Warburg effect, or aerobic glycolysis, is a well-studied process of metabolic redirection that supports the energetic and anabolic requirements of proliferating cells, including psoriatic keratinocytes. Recent studies have reported a significant upregulation of glucose transporter 1 (GLUT1) and key glycolytic enzymes such as phosphoglycerate mutase 1 (PGAM1) and pyruvate kinase 2 (PKM2) in psoriatic epidermal keratinocytes (Zhang et al., 2018; Liu et al., 2021; Zhao et al., 2021). Moreover, pharmacological inhibition or genetic knockout of these glycolytic genes has been shown to arrest keratinocyte proliferation and reduce inflammatory responses effectively (Zhang et al., 2018; Liu et al., 2021; Kim et al., 2022). Mechanistically, hypoxia-inducible factor (HIF)-1α and mammalian target of rapamycin (mTOR) pathways are known to be involved in enhancing glycolysis activity in psoriatic keratinocytes (Kim et al., 2022). Interestingly, HIF-1α/mTOR-mediated glycolysis contributes to the activation and proliferation of lymphocytes as well (Lin et al., 2021), suggesting a potential role for targeting this axis in the suppression of psoriasis progression.

Substantial evidence has indicated that traditional Chinese medicine (TCM) can, to some extent, yield multi-targeted interventions for patients with psoriasis (Kuai et al., 2020; Song et al., 2021). PSORI-CM02 is an optimized formula based on the empirical prescription of yin-liquoring by Professor Xuan Guowei, a national Chinese medicine expert specializing in dermatological diseases. In a real-world study, we found that the oral administration of PSORI-CM02 (also named *Shaoling* formula) for more than 2 months can significantly improve the disease activity of psoriasis. Specifically, the physician’s global assessment (PGA) score decreased from 3.3 ± 1.0 before treatment to 2.8 ± 1.1 after treatment (*n* = 619), body surface area (BSA) decreased from 6.6% ± 9.7% before treatment to 6.3% ± 8.5% after treatment (*n* = 90), psoriasis area and severity index (PASI) decreased from 5.8 ± 3.6 before treatment to 4.4 ± 3.0 after treatment (*n* = 89) (WANG et al., 2021). Our group has conducted several studies on the pharmacodynamic mechanism of PSORI-CM02 in the treatment of psoriasis, revealing its multi-targeted effects in blocking the pathological interaction between keratinocytes and immune cells, such as reducing inflammatory cell infiltration (Li et al., 2020), promoting Treg differentiation (Chen et al., 2018), down-regulating cytokine production (Wu et al., 2019), and inhibiting angiogenesis (Lu et al., 2021). However, the effects of PSORI-CM02 on keratinocytes remain unclear, apart from its ability to induce autophagy of keratinocytes *via* suppressing the phosphatidylinositol 3-kinase (PI3K)/Akt/mTOR pathway (Yue et al., 2019). Given that the mTOR pathway is a crucial player in cell metabolism, it is necessary to understand how PSORI-CM02 reduces keratinocyte proliferation by inducing metabolic reprogramming.

In agreement with our previous study, we find that both pharmaceutic serum and water extracts of PSORI-CM02 inhibited the proliferation of keratinocytes and suppressed the activity of the mTOR pathway. To elucidate the underlying mechanism, we employed Seahorse technology to examine the metabolic effects of PSORI-CM02 on HaCaT cells. Intriguingly, we observed that treatment with the pharmaceutic serum of PSORI-CM02 suppresses glycolysis and glycolytic capacity while simultaneously repressing mitochondrial respiration and ATP production of HaCaT cells. We screened the impact of PSORI-CM02 on a set of glycolytic genes and found HK2 significantly decreased upon treatment of either pharmaceutic serum or water extracts of PSORI-CM02. Additionally, we evaluated the effects of PSORI-CM02 and BPTES, an mTOR inhibitor, on the hyperproliferation of epidermal keratinocytes in the imiquimod-induced psoriasis mouse (IMQ) model. Our results illustrated that treatment with PSORI-CM02 and BPTES reduced the hyperproliferation of epidermal keratinocytes, the number of PCNA-positive cells, and the level of p-S6 in lesioned skin. Taken together, our results provide novel insights into the cellular metabolic mechanisms by which PSORI-CM02 inhibits hyperplasia of psoriatic keratinocytes and highlight the multi-targeted advantages of PSORI-CM02 for the treatment of psoriasis.

## Materials and methods

### Chemical and reagents

Dulbecco’s modified Eagle’s medium (DMEM) (C11995500BT, Gibco, United States) and fetal bovine serum (FBS) (10,099-141, Gibco, United States) were purchased from Gibco Laboratories. BPTES was purchased from Selleck (S7753, Selleck, China). Imiquimod cream was obtained from Sichuan Mingxin Pharmaceutical Co., Ltd., (39210301, Mingxin Pharmaceutical, China). The enhanced chemiluminescence (ECL) reagent was obtained from Millipore (WBKLS0100, Millipore, United States). Polyvinylidene fluoride (PVDF) membrane was purchased from Merck Millipore Ltd. Deionized water was prepared using a Millipore Milli-Q Plus system (Millipore, Bedford, MA, United States). Antibodies specific for p-S6 (S235/236) (4856, CST, United States), S6 (2217, CST, United States), β-actin (12,620, CST, United States), P70S6K (9202, CST, United States), p-P70S6K (9234, CST, United States), PCNA (13,110, CST, United States), and goat anti-rabbit antibodies (7074, CST, United States) conjugated to horseradish peroxidase (HRP) were obtained from Cell Signaling Technology. TRIzol (15596026, Thermo Fisher, United States) was purchased from Thermo Fisher Scientific. HiScript ^®^ III RT SuperMix for qPCR (+gDNA wiper) (R323, Vazyme, China) and ChamQ Universal SYBR qPCR Master Mix (Q711, Vazyme, China) were purchased from Vazyme Biotech Co., Ltd.

### Preparation of PSORI-CM02 water extracts and pharmaceutic serum

We purchased the herbs of PSORI-CM02 made by Guangdong Kangmei Pharmaceutical Co., Ltd., from the Guangdong Provincial Hospital of Chinese Medicine. The PSORI-CM02 water extraction was performed as described previously (Wu et al., 2019; Li et al., 2020). Briefly, the weight ratio of Smilax glabra Roxb., Sarcandra glabra (Thunb.) Nakai, radix of Paeonia lactiflora Pall, radix of Curcuma phaeocaulis Val., and Prunus mume (Sieh.)Sieb. et Zucc., is 5:5:3:2:2. All herbs were dipped in water for 30 min, and heated to keep boiling for 1 h, filtrated by a 75 μm filter. Extracted herbs of PSORI-CM02 using distilled water and repeated twice, and then concentrated and dried out by rotary evaporator under vacuum, stored for use in subsequent experiments at −20°C.

After a three-day acclimation period, 12 SD rats were randomly divided into a vehicle pharmaceutic serum group (VPS) and a PSORI-CM02 pharmaceutic serum group (PPS). Rats in the PPS group received two intragastric injections of PSORI-CM02 per day for 7 days at a dose of 7.2 g/kg, and rats in the VPS group received 2.5 mL saline. 1 h after the last intragastric administration, rats were anesthetized by intraperitoneal injection of 10% chloral hydrate at a dose of 3 mL/kg, and the whole blood was collected, placed at 4°C for 4 h, centrifuged at 3,000 rpm for 25 min. The serums were then inactivated by incubation in a constant temperature water bath at 56°C for 20 min, filtered through 0.22 μm filters, dispensed into EP tubes, and stored at −80°C in the refrigerator. To ensure quality control and stability of PPS, LC-MS/MS analysis was also used to confirm the components in PPS and compare the results with those of PSORI-CM02 water extracts.

### Metabolites extraction and LC-MS/MS analysis

The samples were thawed on ice and subjected to centrifugation at 12,000 rpm (RCF = 13,800 (×g), R = 8.6 cm) for 15 min at 4°C after 30 s of vortexing. A volume of 300 μL of supernatant was collected and transferred to a new tube, followed by the addition of 1,000 μL of extract containing 10 μg/mL of internal standard. The samples were sonicated for 5 min in an ice-water bath and subsequently incubated at −40°C for 1 h. After centrifugation at 12,000 rpm (RCF = 13,800 (×*g*), R = 8.6 cm) for 15 min at 4°C, the supernatant was filtered through a 0.22 μm microporous membrane. In the next step, 400 μL of serum sample was mixed with 40 μL of hydrochloric acid (2 mol/L), vortexed for 1 min, and incubated at 4°C for 15 min. The vortexing and incubation cycle was repeated 4 times. Acetonitrile (1.6 mL) was added to the mixture, which was then vortexed for 5 min and centrifuged at 12,000 rpm (RCF = 13,800 (×*g*), R = 8.6 cm) for 5 min at 4°C. The supernatant (1,800 μL) was transferred to a new tube and dried under nitrogen. The dried sample was reconstituted by vortexing in 150 μL of 80% methanol containing 10 μg/mL of internal standard for 5 min. The sample was then centrifuged at 12,000 rpm (RCF = 13,800 (×*g*), R = 8.6 cm) for 5 min at 4°C, and 120 μL of supernatant was transferred to a fresh glass vial for LC/MS analysis.

LC-MS/MS analysis was conducted using an ultra-performance liquid chromatography system (Vanquish, Thermo Fisher Scientific) equipped with a Waters UPLC BEH C18 column (1.7 μm 2.1*100 mm). The sample injection volume was 5 μL, and the flow rate was set at 0.5 mL/min. The mobile phases consisted of 0.1% formic acid aqueous solution A) and 0.1% formic acid acetonitrile solution B). The multi-step linear elution gradient program comprised 0–11 min, 85%–25% A; 11–12 min, 25%–2% A; 12–14 min, 2%–2% A; 14–14.1 min, 2%–85% A; 14.1–16 min, 85%–85% A. MS and MS/MS data were obtained using the Orbitrap Exploris 120 mass spectrometer and Xcalibur software in the IDA acquisition mode. The acquisition cycle comprised masses ranging from 100 to 1,500, and the first four of each cycle were filtered out, followed by the acquisition of corresponding MS/MS data. The intersheath gas flow rate was 35 Arb, auxiliary gas flow rate was 15 Arb, ion transport tube temperature was 350°C, evaporator temperature was 350°C, full MS resolution was 60,000, MS/MS resolution was 15,000, collision energy was 16/38/42 in NCE mode, and spray voltage: 5.5 kV (positive) or −4 kV (negative).

### Cell culture and animal experiment

HaCaT cells were cultured in DMEM (Gibco) supplemented with 10% FBS and 1% penicillin-streptomycin. The cells were incubated at 37°C in a 5% CO_2_ humidified incubator. HaCaT cells were stimulated with TNF-α (20 ng/mL) for 24 h to induce an inflammatory response.

BALB/c mice (20 g ± 2 g) were purchased from the Center of Laboratory Animals of Southern Medical University (Guangzhou, China) and were raised within a specific pathogen-free (SPF) environment. Ethics approval of all animal experiments was obtained from the Animal Experimental Ethics Committee of Guangdong Provincial Hospital of Chinese Medicine (no. 2018068). The mice were randomly divided into 6 groups (*n* = 5) comprising a control group, an IMQ (saline, oral administration, for 7 consecutive days) group, a BPTES (12.5 mg/kg/day, intraperitoneal injection, for 7 consecutive days) group, and three PSORI-CM02 groups with varying doses (1.2 g/kg, 2.4 g/kg, and 4.8 g/kg for low, medium, and high dose, respectively, oral administration, for 7 consecutive days). The control group was untreated normal mice. The IMQ, BPTES, and PSORI-CM02 groups were induced by applying a 62.5 mg/day dose of 5% IMQ cream on a shaved area (3 cm × 2.5 cm) of the mice’s backs for 7 consecutive days. The PASI scores were measured on the 7th day.

### Western blot analysis

Cells were lysed in sample buffer (0.2% m/v SDS, 10% v/v glycerol, 0.2% v/v β-mercaptoethanol, 60 mmol/L Tris pH 6.8) for protein extraction. Proteins were detected using 7.5%–12.5% SDS-PAGE gels and subsequent Western blot analysis with primary antibodies (1:1,000) detected by HRP-conjugated secondary antibody (1:2500). Unless stated otherwise, β-actin (1:1,000) was used as a loading control.

### Seahorse experiments

Cells were seeded in 6-well plates and treated with 10% VPS or 10% PPS for 24 h. After that, cells were seeded in XF24 polystyrene cell culture microplates (Seahorse Bioscience) at a density of 10,000 cells per well. The Seahorse Bioscience XFe24 Analyzer was employed to measure oxygen consumption rates (OCR) in pmol/min and extracellular acidification rate (ECAR) in mPH/min.

For the mitochondrial stress test, 1 hour before measurement, the culture medium was replaced with Seahorse XF Base medium (Seahorse Bioscience), supplemented with 10 mM glucose (Sigma-Aldrich), 2 mM L-glutamine (Seahorse Bioscience), 5 mM pyruvate (Seahorse Bioscience) and 0.56 μL NaOH (1 M) for the mitochondrial stress test. During the test, 2 μM oligomycin, 1 μM FCCP, and 1 μM of Rotenone and Antimycin A (Seahorse Bioscience) were injected into each well at 18, 45, and 63 min, respectively. All measurement processes and the parameter value calculation followed the protocol of the mitochondrial stress test from Seahorse Bioscience.

For the glycolysis stress test, the culture medium of cells was replaced with Seahorse XF Base medium (Seahorse Bioscience), supplemented with 2 mM L-glutamine and 0.56 μL NaOH (1 M) 1 hour before assay. During the test, 10 mM glucose, 2 μM oligomycin, and 50 mM 2-deoxy-D-glucose (Seahorse Bioscience) were injected into each well after 18, 45, and 63 min, respectively. All measurement processes and the parameter value calculation followed the protocol of the glycolysis stress test from Seahorse Bioscience.

The data from the mitochondrial stress test and glycolysis stress test were normalized to the total amount of protein for each well.

### Immunohistochemistry (IHC) and hematoxylin and eosin (H&E) staining

Skin biopsies were collected from the dorsal area of mice and fixed with 10% neutral buffered formalin (NBF) for more than 48 h. After embedding in paraffin, the samples were sectioned into 4 μm slices and mounted on micro slides. Subsequently, the sections were deparaffinized, heat retrieved by boiling in citrate buffer (pH 6) for 3 min in a pressure cooker and kept at 94°C–96°C for 10 min, then cooled naturally and perforated using 0.2% TBST for 10 min. Following this, the sections were blocked with 3% BSA (Sigma, A9418) and incubated with primary antibodies against PCNA (1:200) and p-S6 (1:200). The immunostaining was performed using an EliVision System-HRP DAB (MXB, DAB-2031). In the end, sections were counterstained with hematoxylin, dehydrated and coverslipped. H&E was conducted under the manufacturer’s instructions. IHC and H&E staining analysis using Image J and based on at least three sections.

### Proliferation measurement

For the colony-forming assay, 2,000 HaCaT cells were seeded in 6-well plates and treated with DMEM medium supplementary with 10% VPS or 10% PPS. The cell culture medium was refreshed every 3 days for 14 days of treatment. To visualize the colonies, cells were washed with PBS twice, fixed with 100% methanol for 20 min, stained with 0.25% Crystal Violet (Sigma, C0775) in 6.25% ethanol for 30 min at room temperature, rinsed in water, and inverted onto the tissue to dry for overnight, and photographed. The stained crystal violets were then dissolved in 10% acetic acid, and the absorbance at 490 nm was measured for quantification. For the Cell Counting Kit 8 (CCK-8) assay, cells were plated in a 96-well microtiter plate and received treatments of 10% VPS, 10% PPS, or different doses of PWE for 24 h. 20 uL of CCK-8 reagent (Yeasen, China) was added to the culture medium and incubated at 37°C for 3 h. Finally, the formazan dye is quantified by measuring the absorbance at 560 nm.

### RNA extraction and quantitative real-time PCR

Total mRNA was extracted following the manufacture of Qiagen RNeasy Kit (Qiagen) and reversed transcribed using the iScript cDNA Synthesis Kit (Bio-Rad). Real-time PCR was performed using SYBR Green FastStart Master Mix (Roche) in the CFX Connect Real-time PCR detection system (Bio-Rad). Data were normalized to beta-actin and presented as relative expression levels, calculated using delta-delta Ct analysis. All primer sequences of targeted genes were derived from the Primerbank (https://pga.mgh.harvard.edu/primerbank/) and listed in Supplementary Table S1.

### Statistical analysis

Data were analyzed with the Student’s *t*-test for two groups comparison, with one-way analysis of variance (ANOVA) for more than three groups comparison, or with two-way ANOVA for multiple comparisons. Data are expressed as the mean ± standard deviation (SD). Statistically significant differences were identified when *p* < 0.05. Statistical analysis was performed using GraphPad Prism 9.0.

## Results

### Analysis of the components of PSORI-CM02 water extract and pharmaceutic serum by LC-MS/MS

**FIGURE 1:**
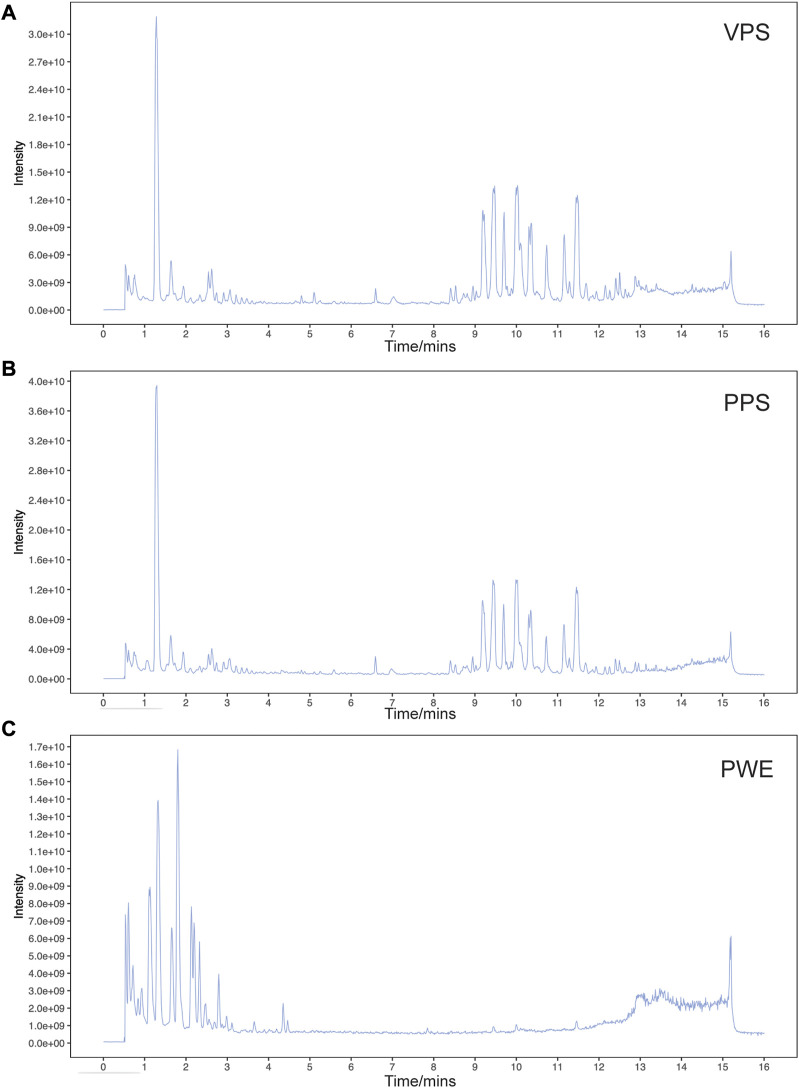
Base peak ion chromatogram of samples analyzed in negative ion mode by LC-MS/MS. Representative chromatogram of vehicle pharmaceutic serum [VPS, **(A)**] PSORI-CM02 pharmaceutic serum [PPS, **(B)**] and PSORI-CM02 water extracts [PWE, **(C)**].

In this study, we used liquid chromatography-tandem mass spectrometry (LC-MS/MS) to detect the components of PSORI-CM02 water extracts (PWE) and its pharmaceutic serum (PPS). Base peak ion chromatograms of vehicle pharmaceutic serum (VPS), PPS, and PWE measured in negative ion mode by LC-MS/MS are presented in Figures 1A–C. A total of 640 compounds were detected after removing duplicate terms, with 276 and 427 compounds detected in the negative and positive ion mode, respectively. Among these compounds, 23 were detected in both PWE and PPS but not in VPS (Table 1), indicating that these compounds can be absorbed into the blood, including 3-hydroxybenzoic acid, ethyl gallate, octyl gallate, piscidic acid, paeoniflorin, beta-asarone, austricine, and isorhamnetin (see Supplementary Table S2 for details). However, the individual or combinatorial biological effects of these compounds need to be clarified and require further investigation.

**TABLE 1:** List of compounds of PSORI-CM02 absorbed into the blood.

No.	Name	Formula	Class
1	Beta-Asarone	C12H16O3	Phenylpropanoids
2	Chaulmoogric Acid	C18H32O2	Fatty acids
3	LOVASTATIN	C24H36O5	Terpenoids
4	Jaeschkeanadiol	C15H26O2	Terpenoids
5	3,8a-Dimethyl-5-methylene-2-oxo-2,4,4a,5,6,7,8,8a,9,9a-decahydronaphtho [2,3-b]furan-8-yl acetate	C17H22O4	Miscellaneous
6	Isorhamnetin	C16H12O7	Flavonoids
7	Sweroside	C16H22O9	Terpenoids
8	Austricine	C15H18O4	Alkaloids
9	Paeoniflorin	C23H28O11	Terpenoids
10	Dihydroresveratrol	C14H14O3	Polyphenols
11	[(1 S)-5-hydroxy-1-[(2S,3R,4S,5S,6R)-3,4,5-trihydroxy-6-(hydroxymethyl)oxan-2-yl]oxy-1,4a,5,7a-tetrahydrocyclopenta [c]pyran-7-yl]methyl benzoate	C22H26O10	Miscellaneous
12	Chenodeoxycholic acid	C24H40O4	Terpenoids
13	Ethyl gallate	C9H10O5	Phenols
14	Spiculisporic acid	C17H28O6	Miscellaneous
15	1,7-bis(4-hydroxyphenyl)heptane-3,5-diol	C19H24O4	Phenylpropanoids and polyketides
16	Xanthyletin	C14H12O3	Phenylpropanoids
17	Piscidic Acid	C11H12O7	Phenylpropanoids and polyketides
18	3-hydroxybenzoic acid	C7H6O3	Benzene and substituted derivatives
19	4-Nitrophenol	C6H5NO3	Phenols
20	Octyl gallate	C15H22O5	Aliphatics
21	Pechueloic Acid	C15H20O3	Terpenoids
22	Scopoletin	C10H8O4	Phenylpropanoids
23	1-[4-hydroxy-3-(3-methylbut-2-enyl)phenyl]ethanone	C13H16O2	Phenylpropanoids and polyketides

### PSORI-CM02 inhibits the proliferation of keratinocytes by suppressing the mTOR pathway

**FIGURE 2:**
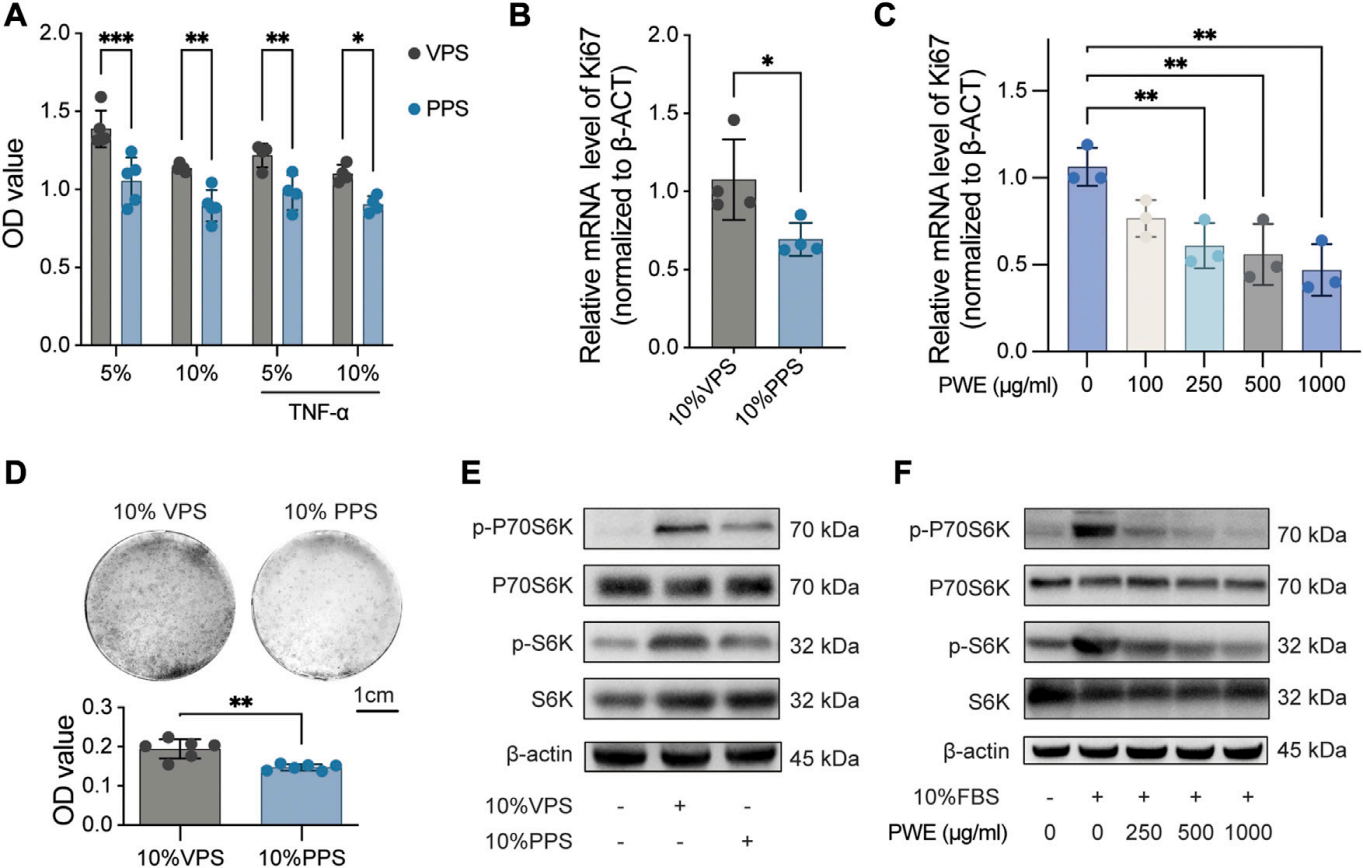
PSORI-CM02 inhibits the proliferation of keratinocytes by suppressing the mTOR pathway. **(A)**. Cell viability of HaCaT cells treated with vehicle pharmaceutic serum (VPS) or PSORI-CM02 pharmaceutic serum (PPS) as assessed by CCK-8 assay. *n* = 4 replicates, *p < 0.05, **p < 0.01, ***p < 0.001. **(B)**. mRNA expression of ki67 in HaCaT cells treated with VPS or PPS as determined by quantitative Real-time PCR assay. *n* = 4 replicates, *p < 0.05. **(C)**. mRNA expression of ki67 in HaCaT cells treated with a range of PSORI-CM02 water extracts (PWE) as determined by quantitative Real-time PCR assay. *n* = 3 replicates, **p < 0.01. **(D)**. Representative images of cell colony-forming assay of HaCaT cells treated with 10% VPS or PPS (upper panel) and the quantification result (down panel). *n* = 6 replicates, ***p < 0.001. **(E)**. Representative blots of p-P70S6K, P70S6K, p-S6, and S6 in HaCaT cells treated with 10% VPS or 10% PPS. β-Actin was loaded as house control. **(F)**. Representative blots of p-P70S6K, P70S6K, p-S6, and S6 in HaCaT cells treated with different doses of PWE. β-Actin was loaded as house control.

To study the effects of PSORI-CM02 in the treatment of psoriasis, we first analyzed the influence of PWE on the proliferative activity of HaCaT cells, a widely used immortalized human keratinocyte line. HaCaT cells were cultured in DMEM medium supplemented with 5% or 10% of VPS and PPS, respectively, for 24 h, and the proliferative activity was determined by CCK-8 assay (Figure 2A). The results indicated that either 5% or 10% of PPS significantly suppressed cell proliferation of HaCaT cells compared to VPS, even under TNF-α-induced inflammatory conditions (Figure 2A). Next, ki67, a well-known proliferation marker, was measured by real-time PCR further to confirm the inhibitory effect of PSORI-CM02 on keratinocyte proliferation. The level of ki67 mRNA was reduced by approximately 30% by PPS compared to VPS (Figure 2B) and decreased by PWE in a dose-dependent manner compared to the vehicle condition (Figure 2C). Furthermore, in a cell colony formation assay, we observed that treatment with PPS resulted in a clear reduction in size and number of cell colonies (Figure 2D). Together, these findings demonstrate that both PWE and PPS negatively affect the proliferation of keratinocytes.

Given that the mTOR pathway is a major contributor to cell proliferation, we analyzed the effects of PWE and PPS on the mTOR pathway. As shown in Figures 2E, F, PPS, and PWE suppressed the phosphorylation levels of S6 and P70S6K (quantification results in Supplementary Figures S1A–D), which are key signaling molecules in the mTOR pathway. In conclusion, our results suggest that PSORI-CM02 inhibits the proliferation of keratinocytes by suppressing the mTOR pathway.

### PSORI-CM02 reduces mitochondrial respiration and glycolysis of HaCaT cells

**FIGURE 3:**
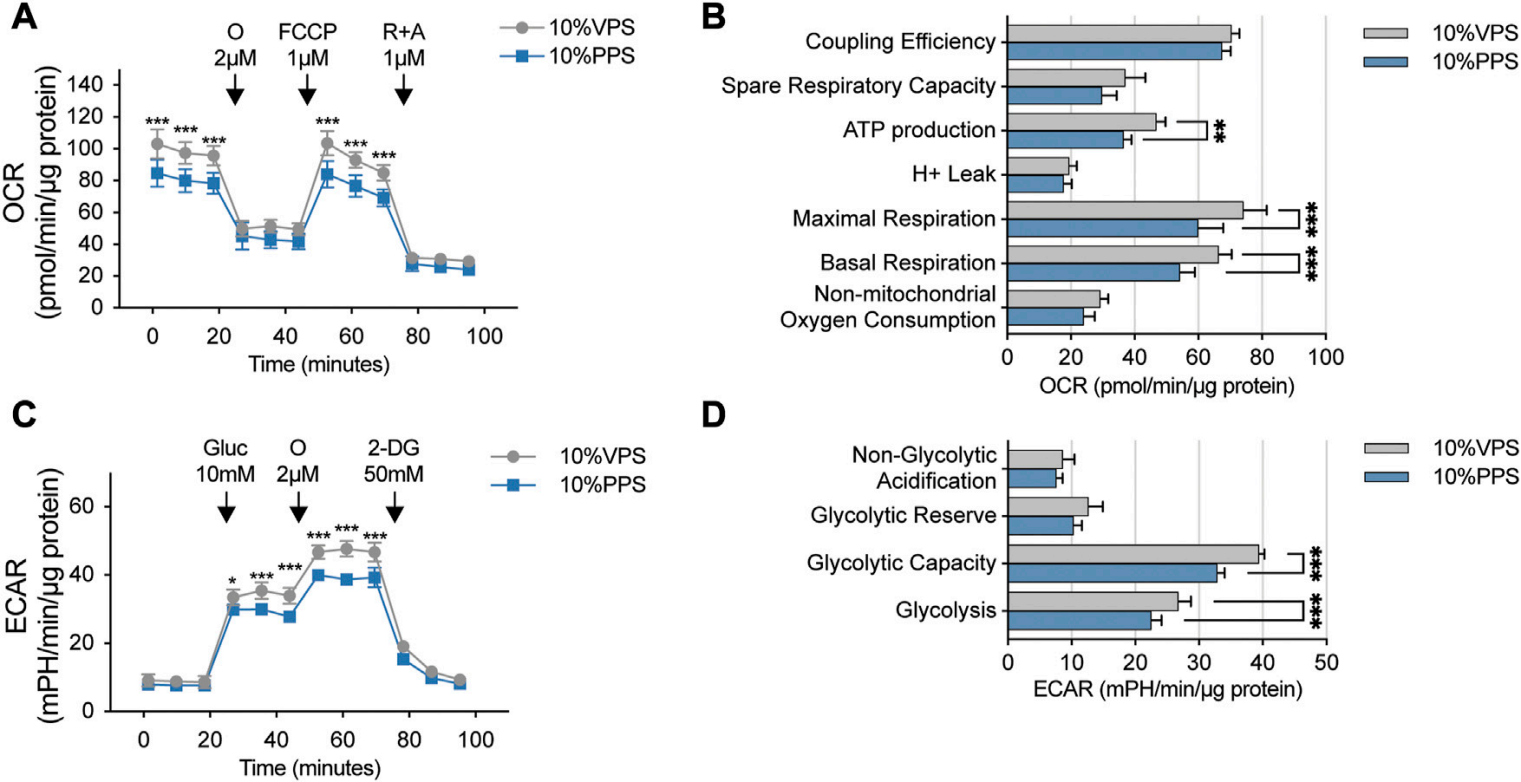
PSORI-CM02 reduces mitochondrial respiration and glycolysis of HaCaT cells. **(A)** Mitochondrial stress test of HaCaT cells upon interventions of vehicle pharmaceutic serum (VPS) and PSORI-CM02 pharmaceutic serum (PPS). OCR, oxygen consumption rate; O, oligomycin; FCCP, Carbonyl cyanide 4-(trifluoromethoxy) phenylhydrazone; R + A, antimycin and rotenone. *n* = 5 replicates, ***p < 0.001. **(B)** Key parameters of mitochondrial stress test from assay showed in A. *n* = 5 replicates, **p < 0.01. **(C)** Glycolysis stress test of HaCaT cells upon treatments of VPS and PPS. ECAR, extracellular acidification rate; Gluc, glucose; O, oligomycin; 2-DG, 2-deoxy-D-glucose. *n* = 5 replicates, *p < 0.05. **(D)** Key parameters of glycolysis stress test from assay showed in C. *n* = 5 replicates, ***p < 0.001.

The mTOR pathway plays a crucial role in regulating cell growth and proliferation by controlling energy metabolism. Thus, we aimed to investigate whether PSORI-CM02 influences either mitochondrial respiration or glycolysis in keratinocytes. We employed Seahorse technology to perform a mitochondrial stress test on HaCaT cells upon treatments of PPS and VPS. As shown in Figures 3A, B, the addition of PPS significantly reduced basal and maximal respiration in HaCaT cells, thus, leading to a decrease in ATP production. Glycolysis is a critical metabolic pathway that produces ATP and provides pyruvate to fuel mitochondrial respiration. Therefore, we also performed a glycolysis stress test assay, which showed that the glycolysis and glycolytic capacity of HaCaT cells were significantly inhibited by PPS (Figures 3C, D). These results demonstrate that PSORI-CM02 has inhibitory effects on cellular energy metabolism, which is in line with its suppression of cell proliferation.

### PSORI-CM02 suppresses glycolysis by reducing the expression of HK2

**FIGURE 4:**
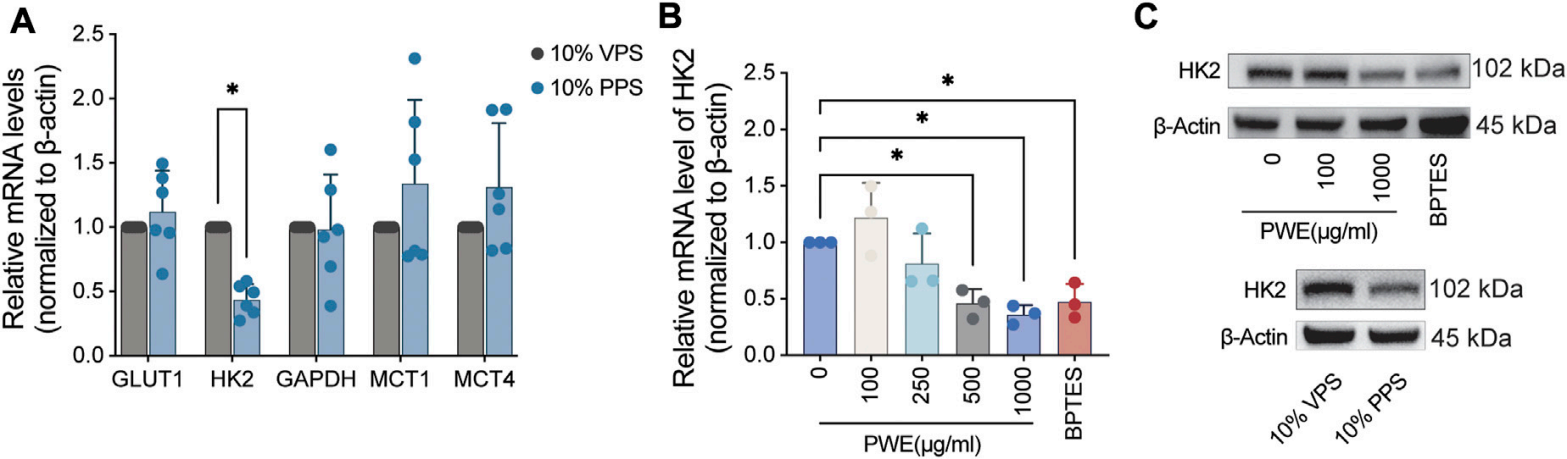
PSORI-CM02 decreases the expression of hexokinase 2 in HaCaT cells. **(A)** Real-time PCR assay of the mRNA levels of glycolytic genes upon treatments of vehicle pharmaceutic serum (VPS) and PSORI-CM02 pharmaceutic serum (PPS). GLUT1, glucose transporter type 1; HK2, hexokinase 2; GAPDH, glyceraldehyde-3-phosphate dehydrogenase; MCT1/4, monocarboxylate transporter 1/4; *n* = 6 replicates, *p < 0.05. **(B)** Real-time PCR assay of the mRNA levels of HK2 upon treatments of PSORI-CM02 water extracts (PWE) and BPTES (an inhibitor of the mTOR pathway). *n* = 3 replicates, *p < 0.05. **(C)** Representative blots of HK2 upon administrations of VPS, PPS, PWE, and BPTES. β-Actin is loaded as house control.

To gain insight into how PSORI-CM02 affects cellular energy metabolism, we focus on the glycolysis pathway as multiple glycolytic genes are regulated by the mTOR pathway, which was repressed by PSORI-CM02 (Figures 2E, F). GLUT1 is the most abundant glucose transporter identified in psoriatic keratinocytes and contributes to the uptake of glucose (Zhang et al., 2018). GLUT1 is a low Km and ATP-independent glucose transporter and hexokinase (HK) mediated glucose-6-phosphate formation is the rate-limiting step for glucose utilization (Ishihara et al., 1994). Glyceraldehyde-3-phosphate dehydrogenase (GAPDH) catalyzes an important energy-yielding step in glycolysis by converting D-glyceraldehyde 3-phosphate into 3-phospho-D-glyceroyl phosphate and generating ATP (Tisdale, 2002). Monocarboxylate transporters (MCT) catalyze the bidirectional proton-linked transport of short-chain monocarboxylates such as L-lactate and pyruvate across the membrane of mammalian cells. MCT1-4 are key in the regulation of many cellular processes like proliferation and inflammation response and act as a good indicator of glycolysis (Hong et al., 2016; Singh et al., 2023). Therefore, we screened the effects of PPS on these five glycolytic genes by real-time PCR and found that PPS significantly inhibited mRNA expression of HK2 (Figure 4A), which mediates the initial step of glycolysis by catalyzing the phosphorylation of D-glucose to D-glucose 6-phosphate (Nawaz et al., 2018). Next, we treated HaCaT cells with a concentration range of PWE and used BPTES as a control. Interestingly, PWE inhibited the expression of HK2 in HaCaT cells in a dose-dependent manner, and high concentrations of PWE showed a similar inhibitory effect on HK2 as BPTES (Figure 4B). To further validate this finding, we treated HaCaT cells with PPS, PWE, or BPTES for 24 h before protein lysate. Consistent with the results at the transcriptional level, we observed that both PPS and PWE significantly downregulated HK2 protein expression (Figure 4C, quantification results in Supplementary Figures S1E, F). In sum, we conclude that PSORI-CM02 inhibits the glycolysis of keratinocytes mainly *via* downregulating the expression of HK2.

### PSORI-CM02 attenuates imiquimod-induced skin thickening in a mouse model of psoriasis

**FIGURE 5:**
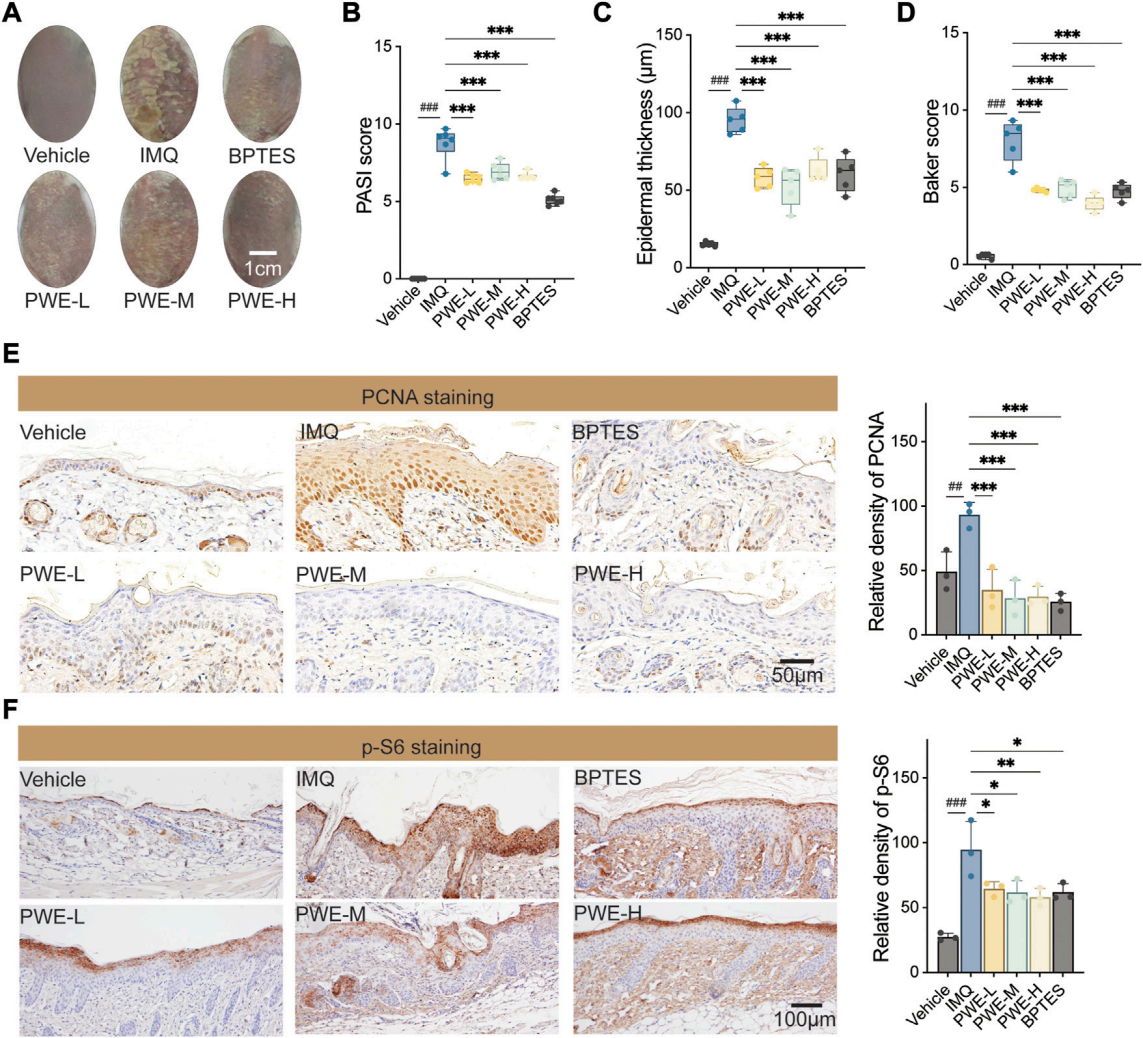
PSORI-CM02 reduces epidermal keratinocytes proliferation and alleviates psoriatic pathology in an imiquimod-induced mouse model. **(A)** Representative images of skin. IMQ, imiquimod-induced psoriatic mouse model; PWE-L/M/H, low-/medium-/high-dose of PSORI-CM02 water extracts; BPTES, an inhibitor of the mTOR pathway. Scale bar: 1 cm. **(B)** The psoriasis area and severity index (PASI) score of mice treated with PWE or BPTES. *n* = 5 replicates, ^###^ *p* < 0.001 vs. vehicle group, ***p < 0.001 vs. IMQ group. **(C)** The epidermal thickness of skin sections from mice upon treatments of PWE or BPTES. *n* = 5 replicates, ***p < 0.001. **(D)** The Baker score of mice upon treatments of PWE or BPTES. *n* = 5 replicates, ***p < 0.001. **(E)** Representative images of immunohistochemistry staining of proliferating cell nuclear antigen (PCNA) in the skin sections of mice treated with PWE or BPTES. Scale bar, 50 μm. Quantification result was presented in the right column, ^##^ *p* < 0.01 vs. vehicle group; ***p < 0.001 vs. IMQ group. **(F)** Representative images of immunohistochemistry staining of p-S6 in the skin sections of mice treated with PWE or BPTES. Scale bar, 100 μm. Quantification result was presented in the right column, ^###^ *p* < 0.001 vs. vehicle group; *p < 0.05 and **p < 0.01 vs. IMQ group.

In order to validate the *in vitro* findings and evaluate the potential *in vivo* effects of PSORI-CM02 mediated inhibition of keratinocyte proliferation, the IMQ mouse model of psoriasis was utilized. Upon topical application of imiquimod, BALB/c mice develop significant psoriasis-like lesion changes, characterized by visibly thickened and covered silvery-white scaly skin surrounded by inflammatory erythema (as depicted in Figure 5A). Oral administration of different doses of PWE resulted in a noticeable attenuation of IMQ-induced skin damage, with effects comparable to those observed with BPTES, an inhibitor of mTOR that served as a positive control in the experiment. In accordance with these observations, treatments with PWE and BPTES significantly reduced the psoriasis area and severity index (PASI) score compared to the IMQ group (Figure 5B). Histological examination by H&E staining revealed that the epidermal thickness of skin sections in the IMQ group was roughly six-fold higher than that of the vehicle group, measuring 95.2 μm ± 8.4 μm and 15.2 μm ± 1.4 μm, respectively. The epidermal thickness of mice administered with different doses of PWE was maintained within the range of 33.5 um–76.8 um, being significantly lower than that of the IMQ group (Figure 5C). Moreover, PWE significantly reduced the IMQ-induced Baker score (Figure 5D), a comprehensive histopathological score of psoriasis that is calculated by taking into account of the number of Munro small cysts, the degree of epidermal keratinization and capillary dilation, the thickness of skin layers, and inflammatory infiltration.

Immunohistochemical staining of skin sections was performed to investigate whether PSORI-CM02 inhibits the hyperproliferation of epidermal keratinocytes induced by IMQ. The representative images depicted in Figure 5E demonstrate that treatments with PWE reduced the number of PCNA-positive keratinocytes, in agreement with the finding that PWE reduced epidermal thickness in Figure 5B. These results confirm our *in vitro* observation that PSORI-CM02 suppresses the proliferation of (psoriatic) keratinocytes. Finally, to access the linkage to mTOR signaling, we also stained p-S6 in skin sections and observed that PSPRI-CM02 had an inhibitory effect on p-S6 comparable to that of BPTES (Figure 5E), consistent with what we found *in vitro* (Figures 2E, F). Altogether, our findings strongly suggest that PSORI-CM02 inhibits keratinocyte proliferation partly by suppressing the mTOR signaling.

## Discussion

Over recent years, aerobic glycolysis has emerged as a new hallmark of proliferating keratinocytes and contributes to the pathogenesis of psoriasis (Zhang et al., 2018). Cells metabolize nutrients for biosynthetic and bioenergetic needs to fuel growth and proliferation. The uptake of glucose from the environment and its intracellular metabolism is a highly controlled process that participates in crosstalk between growth signaling and metabolic pathways. In this context, mTOR, an important signaling molecule, plays a crucial role in integrating growth with metabolism. It responds to extracellular levels of nutrients (glucose and amino acids) and growth signals (Szwed et al., 2021).

In our previous studies, we revealed that the formula of PSORI-CM02 has promising therapeutic effects in the treatment of psoriasis by diminishing the inflammatory response (Chen et al., 2018; Li et al., 2020) and by inhibiting angiogenesis (Lu et al., 2021). However, the impact of PSORI-CM02 on the proliferating keratinocytes, a hallmark of psoriasis, is not well understood. Therefore, in the present study, we focused on the effects of PSORI-CM02 on proliferating keratinocytes and the underlining mechanism. We first found that either PPS or PWE suppressed the proliferation of the immortalized human epidermal keratinocyte HaCaT cells. In addition, both PPS and PWE have strong inhibitory impacts on the activity of the mTOR pathway, in agreement with our previous report (Yue et al., 2019). Accumulating evidence highlights the critical role of the mTOR pathway in promoting aerobic glycolysis (Hu et al., 2020; Weng et al., 2020; Zhang et al., 2022). Therefore, we conducted experiments to test if PSORI-CM02 reduces glycolysis. We observed that PPS diminished the glycolysis and glycolytic capacity while leading to less mitochondrial respiration and ATP production in HaCaT cells. Interestingly, the inhibition of PSORI-CM02 on glycolysis depends on the reduction of HK2 but likely not on other glycolysis-relative enzymes like GLUT1 and GAPDH. HK2 is a downstream enzyme of the mTOR pathway and a rate-limiting step in aerobic glycolysis (Shin et al., 2021; Jiang et al., 2022). Our findings suggest that PSORI-CM02 suppresses keratinocyte proliferation, at least partly, through the mTOR/HK2/glycolysis axis.

In this study, 23 components of PSORI-CM02 absorbed into the blood were identified by mass spectrometry, many of which may have antiproliferative effects and are likely to act in a coordinated manner. Among the identified components, beta-asarone has garnered attention due to its ability to induce autophagy and inhibit cell proliferation in human glioma U251 cells by strongly inhibiting the mTOR pathway (Wang et al., 2018). Additionally, beta-asarone has been shown to induce apoptosis of gastric cancer cells by arresting the cell cycle in the G2/M phase, which is associated with the inhibition of the activity of glycolytic genes, lactic dehydrogenase (LDH), and pyruvate dehydrogenase kinase (PDK) 1 (Tao et al., 2020). Another widely reported component of PSORI-CM02 that blocks the mTOR pathway is isorhamnetin, which is known to repress the proliferation of various tumor cells by inhibiting the PI3K/AKT/mTOR pathway (Li et al., 2014; Hu et al., 2015; Cai et al., 2020). Additionally, isorhamnetin has been reported to reduce glycolysis by inhibiting HIF-1α, thus inhibiting several glycolytic genes, including GLUT1, LDHA, and PDK1 (Seo et al., 2016). Paeoniflorin also can suppress the mTOR pathway and results in the promotion of osteoblast differentiation and mineralization (Yang et al., 2021) and suppression of collagen-induced arthritis (Li et al., 2012). Although evidence is lacking to show that paeoniflorin suppresses cell proliferation *via* the mTOR signal, reports indicate its ability to inhibit cell proliferation (Liu et al., 2022; Wang et al., 2022). These findings suggest that beta-asarone, isorhamnetin, and paeoniflorin, as critical components of PSORI-CM02 that are absorbed into the blood, may act as essential players in the antiproliferative effects of this formula.

Ethyl gallate can directly bind and inhibit the activity of ERK1 and ERK2, thereby inhibiting the growth of esophageal cancer *in vitro* and *in vivo* (Liu et al., 2019); and reducing the activation of PI3K/Akt and nuclear factor-κB (NF-κB), leading to the repression of MDA-MB-231 cells proliferation (Cui et al., 2015). In addition, ethyl gallate is widely reported could induce cell apoptosis by declining the expression of Bcl-2 in different cell lines (Cui et al., 2015; Sanchez-Carranza et al., 2017; Liu et al., 2019). However, whether the repressive effect of ethyl gallate on cell proliferation is dependent on mTOR or glycolysis is not clear yet. Piscidic acid, on the other hand, has yet to be demonstrated to exhibit antiproliferative effects. However, studies have reported that piscidic acid-rich extracts often have better antioxidant and antimicrobial effects, thus benefiting skin health (Petruk et al., 2017) (Alexandre et al., 2021), suggesting that it may, in part, explain the multiple targeted effects of PSORI-CM02. Thus, PSORI-CMO2 harbors several components that in isolation have similar effects on cell signaling and cell proliferation. However, it should be noted that the concentration of the individual components in the PSORI-CM02 formula is likely too low to compare to the concentrations used in previous studies to demonstrate various cellular effects. This supports the notion that within the PSORI-CM02 formula, these compounds act in concert. Unfortunately, the possible function(s) and pharmacological mechanism(s) of most of the remaining identified components of PSORI-CM02 absorbed into the blood are not known yet.

## Conclusion

In sum, we uncovered that PSORI-CM02 exerted an anti-proliferative effect on the psoriatic keratinocytes *in vivo* and *in vitro*. We have further elucidated that the inhibitory effect of PSORI-CM02 on cell proliferation is mediated, at least in part, through the suppression of the mTOR pathway and a reduction in the expression of HK2, which ultimately leads to a decrease in the glycolytic capacity of keratinocytes. Notably, we have identified 23 components of PSORI-CM02 that are absorbed into the blood by mass spectrometry. Although we did not conduct mechanistic studies on the individual components, based on previous reports, beta-asarone, isorhamnetin, and paeoniflorin are likely important contributors to the pharmacodynamic mechanism of PSORI-CM02, given their inhibitory effects on mTOR and glycolysis. This study provides novel insights into the mechanism of action of PSORI-CM02 in treating proliferating keratinocytes in psoriasis.

## Data Availability

The original contributions presented in the study are included in the article/Supplementary Material, further inquiries can be directed to the corresponding authors.

## Supplementary material

SUPPLEMENTARY FIGURE S1 Quantification results of Western blots. **(A)** Quantification and statistical analysis of Western blots of p-P70S6K and P70S6K in Figure 2E. *n* = 3 replicates, ### p<0.001, 10% VPS vs. 0% FBS; *p<0.05, 10% PPS vs. 10% VPS. **(B)** Quantification and statistical analysis of Western blots of p-S6 and S6 in Figure 2E. *n* = 3 replicates, ### p<0.001, 10% VPS vs. 0% FBS; ***p<0.001, 10% PPS vs. 10% VPS. **(C)** Quantification and statistical analysis of Western blots of p-P70S6K and P70S6K in Figure 2F. *n* = 3 replicates, ### p<0.001, 10% VPS vs. 0% FBS; *p<0.05, **p<0.01, ***p<0.01, vs. 10% FBS. **(D)** Quantification and statistical analysis of Western blots of p-S6 and S6 in Figure 2F. *n* = 3 replicates, ### p<0.001, 10% VPS vs. 0% FBS; *p<0.05, **p<0.01, ***p<0.01, vs. 10% FBS. **(E)** Quantification and statistical analysis of Western blots of HK2 and β-actin in upper panel of Figure 4C. *n* = 3 replicates, *p<0.05. **(F)** Quantification and statistical analysis of Western blots of HK2 and β-actin in bottom panel of Figure 4C. *n* = 3 replicates, **p<0.01.
