# Supplementary figures and images for: Deciphering the mechanism of PSORI-CM02 in suppressing keratinocyte proliferation through the mTOR/HK2/glycolysis axis
Source: Front Pharmacol. 2023 Apr 7;14:1152347. doi: 10.3389/fphar.2023.1152347 (PMC10119413; doi:10.3389/fphar.2023.1152347)

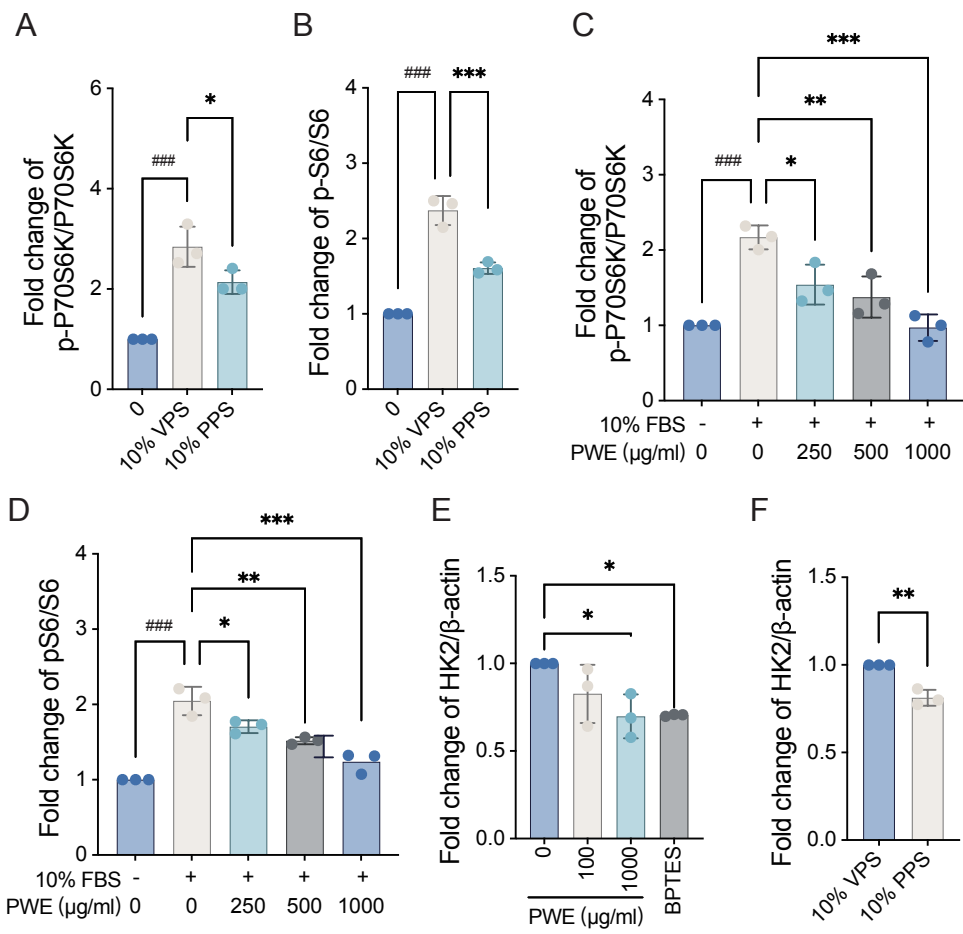

Supplement: Supplementary file 3 [file Image1.pdf]
